# Neuroblastoma in the Era of Precision Medicine: A Clinical Review

**DOI:** 10.3390/cancers15194722

**Published:** 2023-09-26

**Authors:** Andrew Wahba, Russ Wolters, Jennifer H. Foster

**Affiliations:** Department of Pediatrics, Baylor College of Medicine, Texas Children’s Cancer Center, Houston, TX 77030, USA; aawahba@texaschildrens.org (A.W.); rjwolter@texaschildrens.org (R.W.)

**Keywords:** neuroblastoma, pediatric oncology, precision medicine

## Abstract

**Simple Summary:**

Patients with high-risk neuroblastoma, especially those whose disease either does not respond or recurs, have a poor chance of survival. Additionally, the majority of survivors of modern day high-risk neuroblastoma therapy develop significant late-effects. In an effort to improve survival, new agents with less toxicity are being explored. As technologies advance, the ability to design therapies for specific molecular targets on neuroblastoma are crucial. The most notable existing targeted therapies for high-risk neuroblastoma thus far are ALK inhibitors and anti-GD2 therapies, both of which have become mainstays of treatment both in the upfront and relapsed settings. Development of additional molecular targeted therapy has proven challenging, with limited successes. Adding to this difficulty is the heterogeneity of high-risk neuroblastoma, especially in the relapsed setting. This heterogeneity makes the numbers of patients eligible for specific targeted therapy small, complicating optimal clinical trial design. Here, we review the known molecular targets of neuroblastoma and their clinical implications.

**Abstract:**

The latest advances in treatment for patients with neuroblastoma are constantly being incorporated into clinical trials and clinical practice standards, resulting in incremental improvements in the survival of patients over time. Survivors of high-risk neuroblastoma (HRNBL), however, continue to develop treatment-related late effects. Additionally, for the majority of the nearly 50% of patients with HRNBL who experience relapse, no curative therapy currently exists. As technologies in diagnostic and molecular profiling techniques rapidly advance, so does the discovery of potential treatment targets. Here, we discuss the current clinical landscape of therapies for neuroblastoma in the era of precision medicine.

## 1. Introduction

Neuroblastoma is a unique disease with diverse behavior that ranges from spontaneous regression to aggressive metastatic spread. It is the most common pediatric extra-cranial solid tumor, accounting for up to 10% of cancer cases and 15% of cancer deaths in children [1].

High-risk neuroblastoma (HRNBL) remains a challenging cancer to treat. The management paradigm for HRNBL has evolved to now include surgical resection, cytotoxic chemotherapy, autologous stem cell transplant, immunotherapy, isotretinoin, and radiation therapy. Despite this intensive multipronged approach, 3-year event-free survival has not surpassed 65%, and many children will either progress through induction therapy or eventually relapse [2]. The vast majority of patients with relapsed HRNBL are not able to achieve cure. In addition, this intensive therapy is highly toxic and can result in significant long-term morbidity. These dismal outcomes demonstrate the urgent need to leverage precision medicine to identify actionable genomic aberrations and mutations. The precision medicine approach to pediatric oncology has been described in many recent studies, most notably the National Cancer Institute (NCI) Children’s Oncology Group (COG) Pediatric MATCH trial [3,4]. Pediatric MATCH was a Phase 2 trial for pediatric patients with relapsed or refractory tumors. The trial evaluated pre-selected study drugs, each targeting a defined set of genomic mutations. Patients were matched with therapies specific to the molecular abnormalities in their tumor. Of the first 1000 patients screened, 60 (6%) had neuroblastoma. Notably, 45% of these tumors had an actionable mutation of interest. Similar studies using sequencing technologies to identify molecular targets include the German Individualized Therapy for Relapsed Malignancies in Childhood (INFORM) project, the Precision in Pediatric Sequencing (PIPseq) program at Columbia University Medical Center, the Individualized Cancer Therapy (iCAT) Study at Dana-Farber Cancer Institute/Boston Children’s Hospital, the KidsCanSeq and BASIC3 projects at Texas Children’s Hospital, and the Pediatric Michigan Oncology Sequencing (PEDS-MIONCODEQ) study. By characterizing the molecular profile of high-risk neuroblastoma and identifying scientifically rational targets, the hope is to find more effective and less toxic therapies for children with this aggressive disease. This paper will describe the current clinical trial landscape, progress, and the challenges of using these molecular targets to treat HRNBL.

## 2. Molecular Targets

Two molecular targets are already being utilized in the upfront treatment of HRNBL: GD2 is targeted by the monoclonal antibody dinutuximab, and anaplastic lymphoma kinase (*ALK*) is targeted by the tyrosine kinase inhibitor lorlatinib in the current upfront COG study for patients with HRNBL, ANBL1531 (NCT03126916). Other potential molecular targets have been identified, including *MDM2*, *Aurora Kinase*, *B7H3*, *CD46*, *MTOR, BET*, *MEK*, and *RAS* (Table 1). Though *MYCN* would appear to be a promising target given its prevalence and importance in HRNBL, directly targeting *MYCN* amplification and MYCN protein expression has proven challenging. A list of FDA-approved molecular targets that are currently in trials for patients with HRNBL are listed in Table 2.

### 2.1. Anaplastic Lymphoma Kinase (ALK)

Anaplastic lymphoma kinase (ALK) is a promising target for HRNBL. ALK aberrations (mutations or amplifications) are present in most cases of hereditary neuroblastoma [5] and in 10–15% of sporadic cases. The prevalence of ALK aberrations is even higher in relapsed and metastatic neuroblastoma [6,7]. ALK amplification, in particular, appears to be associated with poor outcomes. The Children’s Oncology Group pilot trial ANBL12P1 (NCT01798004) demonstrated the logistical feasibility of obtaining ALK status within 21 days of receiving a tumor sample. Studies have also demonstrated the ability to identify ALK mutations with targeted next-generation sequencing [3]. The tyrosine kinase inhibitor (TKI) Crizotinib has shown to be effective in treating ALK-aberrant neuroblastoma [8]. ALK-targeted therapies have not been without side effects. Notable adverse effect of crizotinib include mouth sores, prolonged QT interval, edema, visual disturbances, interstitial pulmonary disease, and an increased risk of thrombotic events.

In patients with ALK amplification enrolled on ADVL0912, crizotinib did not produce a response. The authors theorized that this may have been due to the inability to administer sufficient concentrations of crizotinib to overcome the competing ATP affinity. This trial did find that crizotinib had activity against R1275Q-mutated HRNBL [8]. A specific mutation in ALK, F1174L, causes resistance in neuroblastoma cells to crizotinib. This resistance may be overcome by giving crizotinib at higher doses [9]. Based on the tumor responses in subgroups of patients, crizotinib was incorporated into the most recent COG study ANBL1531 for patients with ALK-aberrant tumors. It has since been replaced by the third-generation *ALK* inhibitor lorlatinib. Lorlatinib is a TKI that has promise both in vitro and in vivo to overcome drug-resistant *ALK* variant. In addition, compared to crizotinib, lorlatinib has better central nervous system (CNS) penetration. This is an important benefit for a disease like neuroblastoma that can metastasize to the brain and spinal cord. The New Approaches to Neuroblastoma Therapy Consortium (NANT) is conducting a phase 1 study of lorlatinib for children with *ALK*-mutated refractory or relapsed neuroblastoma (NCT03107988). Because of the encouraging results from this Phase 1 study showing single agent response rates of 30%, and response rates up to 48% in combination with chemotherapy, lorlatinib has been incorporated into the current COG protocol ANBL1531 (NCT03126916), replacing crizotinib [10]. Within this study, patients whose tumors harbor an ALK aberration were non-randomly assigned to receive lorlatinib combined with COG standard therapy for HRNBL during all phases of treatment (induction, consolidation, post-consolidation) as well as a continuation phase. Notable adverse effects are CNS effects (seizures, psychotic events, mood changes, sleep changes), hypercholesterolemia, and weight gain.

Additional second-generation ALK inhibitors, including alectinib, ceritinib and ensartinib are currently being evaluated in early phase trials for patients with relapsed HRNBL. Alectinib has shown promising results both in small case series as well as pre-clinically [11,12]. Similarly, in phase 1 trials, patients treated with ceritinib had an overall response of 20% [13]. Entrectinib, a TRKA/B/C, ROS1, and ALK tyrosine kinase inhibitor, however, did not have the same promising results, with an overall response of 6% [14]. Repotrectinib, a next=generation TRK/ROS1/ALK inhibitor, is currently being studied. Given the propensity of ALK-mutated neuroblastoma to develop ALK resistance, maintaining a pipeline of ALK inhibitors with the ability to overcome this resistance remains an important area of research.

### 2.2. Aurora Kinase

Aurora kinases are a family of serine/threonine kinases. These kinases function as regulators of mitosis. They are subdivided into three distinct groups: A, B and C [15]. Expression and amplification of Aurora kinase A is a negative prognostic marker. Specifically, overexpression of Aurora A mRNA in neuroblastoma tumor tissue was shown to be associated with high-risk disease, higher stage, unfavorable histology, MYCN amplification (*p* = 0.017), disease relapse (*p* = 0.019), and decreased progression-free survival [16]. Amplification of MYCN is a significant negative risk factor in neuroblastoma [17]. However, directly targeting MYCN amplification and MYCN protein expression has proven difficult. Indirectly influencing MYCN through Aurora Kinase is one potential targeting strategy. Aurora kinase A forms a complex with MYCN, thereby stabilizing it [18]. Preclinical data have suggested the efficacy of the Aurora kinase A inhibitors alisertib and erbumine [19,20]. Early phase clinical trials of Alisertib plus irinotecan and temozolomide demonstrated encouraging antitumor activity [21]. A Phase 2 trial has evaluated the regimen of the Aurora Kinase A inhibitor alisertib (MLN8237) 60 mg/m^2^/dose on days 1 to 7, irinotecan 50 mg/m^2^/dose IV on days 1 to 5, and temozolomide 100 mg/m^2^/dose PO on days 1 to 5 for children with relapsed or refractory NBL. A separate cohort received an oral solution formulation of alisertib (45 mg/m^2^/dose). Partial responses were observed in 19 evaluate patients (21%) with a median 1-year PFS of 34%. When the data were pooled for the phase I and II cohorts, MYCN non-amplified tumors fared much better than MYCN amplified tumors, with a 1-year PFS of 47% (CI 37–57%) compared to 10% (CI 1–19%) [22]. This response was better than expected based on historical cohorts treated with irinotecan and temozolomide alone [23]. Alisertib has been well tolerated, with the most common side effects being anemia and neutropenia.

### 2.3. MDM2

Preclinical studies have demonstrated overexpression of the murine double minute 2 (MDM2) oncogene in some neuroblastoma cell lines. This overexpression of MDM2 leads to inhibition of the important tumor suppressor gene TP53, as well as overexpression of MYCN and VEGF mRNA. Enhanced MDM2 expression has been associated with worse prognosis in other malignancies. Based on these findings, therapeutic strategies are under investigation to restore p53 function by interfering with its interaction with MDM2 [24]. One study tested a selective MDM2 inhibitor, SP141, for efficacy in neuroblastoma tumor models. In both cell culture and in animal models, SP141 demonstrated an anti-tumor effect. The authors note that SP141 inhibits MDM2 via p53-dependent and p53-indepenent mechanisms [17]. Clinical trials assessing MDM2 inhibition in neuroblastoma are ongoing (NCT03654716, NCT03611868).

### 2.4. GD2

Disialoganglioside (GD2) is a glycolipid expressed on the surface of most neuroblastoma cells and is considered a cornerstone of HRNBL therapy. GD2 is also expressed in normal tissue, but it is limited to the central nervous system, peripheral nerves, melanocytes, and mesenchymal stem cells. GD2 is recognized as one of the top cancer antigens/targets of the National Cancer Institute pilot project in 2009, and is also detected in bone and soft tissue sarcomas, melanoma, and retinoblastoma [25,26,27]. In the United States, two anti-GD2 monoclonal antibodies (Dinutuximab and Naxitamab) have proven to be effective in treating patients with neuroblastoma, and were approved by FDA in 2015 and 2020, respectively.

Dinutuximab (ch14.18) is a novel chimeric human-murine monoclonal antibody that binds to surface GD2, leading to antibody-mediated cytotoxicity. Early clinical trials in pediatric oncology showed that the main toxicities, both alone and in combination with GM-CSF and IL-2, were severe neuropathic pain, capillary leak syndrome, hypotension, and fever [28,29]. Those early phase trials were the foundation of the pivotal COG phase III clinical trial (NCT00026312), randomizing post-consolidation HRNBL patients to receive either standard therapy (six cycles of isotretinoin) or immunotherapy (six cycles of isotretinoin and five cycles of dinutuximab in combination with alternating GM-CSF and IL-2). The dinutuximab arm was found to be superior, with a 2-year event-free survival (EFS) of 66% vs. 46% (*p* = 0.01) and an overall survival (OS) of 86% vs. 75% (*p* = 0.02). All subsequent patients were treated with dinutuximab, GMCSF, and IL-2, with a 5-year EFS of 73.2% vs. 56.6% (*p* = 0.045) [30,31]. Similar to the early phase trials, neuropathic pain was the most reported toxicity, followed by fever, capillary leak syndrome, hypersensitivity reaction, electrolyte abnormality, and transaminitis. Based on subsequent trials showing no benefit of the addition of IL-2, the standard of care in North America for HRNBL in post-consolidation is dinutuximab and GM-CSF. In Europe, dinutuximab beta (ch14.18/CHO) is used instead of dinutuximab, with comparable outcomes [32,33,34].

Dinutuximab, in combination with irinotecan and temozolomide (DIT), is also effective in patients with relapsed or refractory HRNBL. A randomized COG phase II clinical trial (NCT01767194) for patients with relapsed or refractory HRNBL compared the response and toxicity for those treated with DIT combined with GM-CSF vs. temsirolimus (mTOR). Some 53% of the patients that received DIT had partial to complete response, compared to only 6% for those treated with temsirolimus [32]. Another 36 patients were non-randomly assigned to the DIT arm, 36% of whom had a partial-to-complete response. One-year progression-free survival was 67.9%, and overall survival was 84.9% [33]. Due to its proven efficacy in relapsed or refractory HRNBL, DIT has become the standard of care for these patients. Given its success in combination with irinotecan and temozolomide, other novel combinations are being evaluated in clinical trials, including I-131 MIBG, eflornithine, abemaciclib, nivolumab, NK cells, lenalidomide, and vorinostat.

Naxitamab (hu3F8) is a humanized (IgG1) anti-GD-2 monoclonal antibody. It is administered over 30–60 min in the outpatient setting, compared to dinutuximab, which is given over a minimum of 10 h as an inpatient. Ongoing phase II trials (NCT03363373) have shown that Naxitamab can be effective in patients with relapsed HRNBL limited to bone/bone marrow who have previously responded to or have stable disease from prior therapy [35]. Naxitimab is also currently being investigated in the upfront treatment setting (NCT05489887).

Given the effectiveness of anti-GD2 therapy in HRNBL, novel anti-GD2 therapies are being developed.

### 2.5. B7H3

B7-H3 (B7 homolog 3 protein) (CD 276), an immune checkpoint molecule that is widely expressed on activated T cells, monocytes, and dendritic cells, is overexpressed in multiple cancer types, thus making it a promising immunotherapy target [34,36,37]. The B7-H3 gene is located on chromosome 15 (15q24.1) and can be expressed as a type I transmembrane glycoprotein. Cancer overexpression of B7-H3 has been associated with worse prognosis and higher recurrence rates [36,38,39,40]. Three potential B7-H3 counter-receptors have been identified: triggering receptor expressed on myeloid cells (TREM) like transcript 2 (TLT-2), interleukin 20 receptor subunit α (IL20RA), and phospholipase A2 receptor 1 (PLA2R1) [36,41]. TLT-2 was the first counterreceptor to be identified. It is expressed on CD8+ T cells, activated CD4+ T cells, B cells, neutrophils, and macrophages. Previous reports have shown that B7-H3–TLT-2 interaction enhances T-cell activation, especially CD8+ cells [42]. B7-H3 interaction with IL20RA or PLA2R1 is not well established, and further in vivo and in vitro studies are needed to help better understand those interactions [36].

B7-H3 is widely expressed in NBL tissue and shows increased mRNA expression in advanced NBL stages [43]. A recent study showed that 17 out of 18 patients with NBL had positive B7-H3 expression, with 14 patients (82%) showing moderate-to-strong positive expression. Another study involving 1864 NBL patients showed that strong B7-H3 expression was associated with worse overall survival (*p* < 0.05) [40].

In recent years, novel immunotherapy agents targeting B7-H3 have been developed. [36,38,43,44,45]. Enoblituzumab (MGA271), an investigational humanized monoclonal antibody targeting B7-H3, has recently completed a Phase 1 trial in children with solid tumors expressing B7-H3, and Vobramitamab duocarmazine (MGC018), a B7-H3 monoclonal antibody conjugated to a DNA-alkylating agent (Duocarmycin), is currently in early-phase adult clinical trials both alone and in combination with pembrolizumab (NCT02982941, NCT02475213, NCT03729596) [46,47].

131I-Omburtamab (8H9), an anti B7-H3 investigational radiolabeled murine monoclonal antibody, is currently being studied for localized central nervous system delivery, including in HRNBL patients with leptomeningeal metastases (NCT00089245). Overall injections were well tolerated, with myelosuppression being the most common side effect. Median progression-free survival in HRNBL patients was 7.5 years [48]. NCT03275402 is a currently open phase II/III clinical trial assessing the efficacy of 131I-omburtamab in HRNBL patients with CNS/leptomeningeal metastases. Based on these clinical trials, in 2022, 131I-Omburtamab submitted a biologics license application to the FDA for pediatric patients with HRNBL CNS/leptomeningeal metastases; however, it was rejected based on an inability to reliably assess responses in treated patients.

B7-H3 remains a promising target with multiple ongoing preclinical and clinical trials.

### 2.6. CDK 4/6 Inhibitors

Another potential target is the cell cycle regulator cyclin-dependent kinase (CDK) 4/6. Stimulating CDK 4/6 causes phosphorylation (inactivation) of retinoblastoma (RB) tumor suppression protein, which promotes cellular progression and proliferation [49,50]. Several CDK 4/6 inhibitors are currently being investigated, including palbociclib, ribociclib, and abemaciclib [51]. CDK 4/6 was found to be over-expressed in neuroblastoma, with robust phosphorylation of RB [50]. Phase I trials showed that CDK 4/6 inhibitors have an acceptable toxicity profile, with drug-induced leukopenia/neutropenia being the most reported adverse event (72%). Non-hematologic toxicities included vomiting, fatigue, and QTC prolongation [52,53]. A portion of patients treated with ribociclib showed prolonged stable disease [52]. In NBL preclinical studies, a synergistic growth inhibition effect was noted when combining CDK 4/6 and ALK inhibitors [54]. Ongoing clinical trials are assessing their safety and efficacy in combination with traditional salvage regimens including cyclophosphamide/topotecan (NCT03709680), dinutuximab/irinotecan/temozolomide (NCT04238819), and topotecan/temozolomide (NCT05429502).

### 2.7. RAS-MAPK Inhibitors

The MAPK pathway, a promoter of cell proliferation, leads to the activation of multiple downstream pathways including MEK, ERK, RAF, and RAS [55]. Patients with relapsed HRNBL can show over-activation of the MAPK pathway compared to patients at initial diagnosis, making it a potential therapeutic target [24]. Additionally, as this pathway is downstream from ALK, targeting ALK may also have an impact. Pre-clinical evaluations and early-phase trials of ERK, RAS and MEK inhibitors are currently underway [56]. In Phase 2 trials with selumetinib and ulixertinib for pediatric patients with MAPK pathway alterations, no objective responses were observed [57]. Current strategies are focused on ways to combine these inhibitors in order to improve efficacy.

### 2.8. mTOR Inhibitors

The mammalian target of rapamycin (mTOR) is a protein kinase, part of the phosphoinositide 3-kinase (PI3K)-related kinase family, which plays a vital role in regulating protein synthesis and tumor progression [58,59]. Preclinical studies showed aberrant activation of the PI3K/Akt/mTOR pathway in NBL tissue samples, and mTOR inhibitors had an anti-proliferative effect on NBL cells, especially with cells expressing high levels of MYCN [60,61,62]. Multiple mTOR inhibitors (sirolimus, temsirolimus (TEM), everolimus) are currently being investigated in HRNBL. Although a Phase 2 trial of TEM in combination with irinotecan and temozolomide showed only a 6% response rate [32], future studies are focused on combining PI3K/Akt/mTOR pathway target agents to produce a synergistic effect and potentially improve the antitumor activity in NBL.

## 3. Chimeric Antigen Receptor T Cells (CAR T Cells)

CAR T cells are effective in treating refractory leukemia, but that success has unfortunately not yet been replicated with solid tumors. Although NBL has many promising targets for CAR T therapies (GD2, L1-CAM, GPC2, B7/H3 and ALK), effectiveness is still limited by heterogeneous antigen expression, T cell exhaustion, and bulky disease. Current studies are focused on exploring strategies to overcome these obstacles.

CAR T cells targeting GD2 were safely administered to HRNBL patients without significant toxicity or neuropathic pain. First-generation GD2-CAR T cells showed some anti-tumor effect but were limited by expansion, Multiple newer generations are currently being investigated with varying results [38,39,40]. A recent promising trial of GD2-CAR T cells expressing the inducible caspase 9 suicide gene (GD2-CART01) showed an overall response rate of 63% in heavily pretreated patients with HRNBL [40]. Novel natural killer T cell (NKT) CARs have also shown promising results in early phase trials, with a response rate of 25% achieved and no dose-limiting toxicity or maximum tolerated dose reached [41]. CAR T and NK cells targeting B7-H3 expressed NBL cells have demonstrated promising results in the preclinical settings [56,57]. Glypican-2 (GPC2) is an antigen expressed during the early phases of fetal development and also expressed in NBL. Its expression, however, is lower than that of its counterparts, GD2 and B7H3 [58]. Optimization of the GPC2 CAR showed promising pre-clinical results, leading to the development on an ongoing Phase 1 trial (NCT05650749) [59]. In an effort to try to overcome heterogeneous expression, bicistronic or dual targets (GPC2 and B7H3) CAR T cells are being investigated [63]. L1-CAM (L1 cell adhesion molecule) (CD171) is another antigen that is overexpressed in NBL cells, and first-generation CAR T cells showed a partial response in a patient with NBL who had a limited disease burden [64]. In an ongoing phase 1 trial (NCT02311621) investigating the safety of second- and third-generation CD171-specific CAR T cells, transient skin rash and hyponatremia have been reported as the potential main side effects.

## 4. Conclusions and Future Directions

Neuroblastoma is a complex tumor with different molecular and genetic features affecting risk stratification, prognosis, and the development of potential new treatments. Despite recent clinical advances, HRNBL remains a challenging tumor to treat, especially for patients with relapsed disease. Additionally, the intensity of therapy has a high risk of leaving survivors with potentially debilitating late effects including ototoxicity, infertility, endocrinopathies, and renal, cardiac, and pulmonary toxicity [65]. Molecular targets thus offer the potential to maintain or improve survival while decreasing the late effects of cytotoxic therapy.

As illustrated above, results from preclinical studies are often not reproduced in clinical practice. Patient-derived xenografts (PDX) are an important strategy to bridge preclinical findings to relevant clinical applications. PDX models are especially useful in pediatric oncology because these diseases are rare, resulting in statistical challenges when designing clinical trials. Compared to single cell lines, PDX models can better mimic the complexities and heterogeneity of pediatric solid tumors and the tumor microenvironment. In addition, the development of metastases can be evaluated to identify promising therapeutic targets [66]. PDX models for neuroblastoma have been created in immunocompromised mice [67]. Multiple studies have demonstrated that these PDX models are able to retain the features of neuroblastoma tumors that are essential for translational research [68]. The limitations of PDX models include the limited supply of tumor samples in rare cancers, the potential for a different tumor microenvironment in murine models, the growth of murine-derived tumors at the PDX site, possible murine-specific tumor evolution, and the difficulty of conducting in vivo studies [69]. In spite of these challenges, PDX models represent a promising mechanism for efficient translational research in HRNBL [70,71,72,73].

In this article, we have discussed several promising targeted agents for NBL, both in clinical practice and in the early stages of development. Additionally, this article highlights the pitfalls of relying on single-agent data to determine if there is adequate anti-tumor activity warranting further exploration. Many of the pathways described above are promising targets in HRNBL. Combining targeting mechanisms to maximize anti-tumor effects is a key next step in exploring these agents. Understanding overlapping toxicities and potential pharmacokinetic influences will be critical considerations in order to safely combine agents. The upcoming ComboMATCH trial will thus be an excellent mechanism for testing combinations of targeted agents (NCT05564377). An ideal molecular target would, like GD2, be present on the majority of neuroblastomas. Given the heterogeneity of HRNBL, especially in the relapsed setting, agents that target a subset of neuroblastoma such as *ALK* inhibitors will also play a critical role. Designing clinical trials for these agents, however, becomes increasingly hard, as the number of patients with neuroblastoma expressing these potential targets are few. The most notable progress in targeted treatment for HRNBL includes *ALK* inhibition and anti-GD2 therapies. These therapies underwent decades of pre-clinical and early phase clinical evaluations before incorporation into front-line trials, highlighting the need for more efficient identification of molecular targets and subsequent clinical translation.

## Figures and Tables

**Table 1:** Active molecularly targeted clinical trials for pediatric patients with high-risk neuroblastoma.

Molecular Target	Agent	Active Clinical Trial	Upfront or Relapsed/Refractory
ALK	Crizotinib	NCT01121588	Both
	Lorlatinib	NCT03107988, NCT03126916	Both
	Alectinib	NCT05770037	Relapsed/refractory
	Ceritinib	NCT05489887, NCT02559778	Relapsed/refractory
	Ensartinib	NCT03213652	Relapsed/refractory
TRK/ROS1/ALK	Entrectinib	NCT02650401, NCT04589845	Relapsed/refractory
	Reprotrectinib	NCT04094610, NCT03093116, NCT03093116	Relapsed/refractory
Aurora kinase A	Erbumine	NCT04106219	Relapsed/refractory
MDM2	ALRN-6924	NCT03654716	Relapsed/refractory
	APG-115	NCT03611868	Relapsed/refractory
GD2	GD2-CART01	NCT03373097	Relapsed/refractory
	iC9-GD2 T	NCT01822652	Relapsed/refractory
	C7R-GD2.CART	NCT03635632	Relapsed/refractory
	BCD-245	NCT05782959	Relapsed/refractory
	iC9.GD2.CAR.IL-15 T	NCT03721068	Relapsed/refractory
	GINAKIT	NCT03294954	Relapsed/refractory
	Ex Vivo Expanded Allogeneic γδ T Cells	NCT05400603	Relapsed/refractory
	Naxitimab	NCT05489887, NCT03363373, NCT02650648, NCT01419834	Both
	Dinutuximab beta	NCT02914405, NCT01704716,,,,,NCT05272371, NCT04221035, NCT05754684	Both
	Dinutuximab	NCT03332667, NCT03794349, NCT04211675, NCT03126916, NCT02573896	Both
GPC2	GPC2 CAR T	NCT05650749	Relapsed/refractory
B7H3	131I-Omburtamab	NCT04022213	Relapsed/refractory
	B7H3 CAR T	NCT04483778	Relapsed/refractory
RAS-MAPK	Selumetinib	NCT03213691	Relapsed/refractory
	Binimetinib	NCT05564377	Relapsed/refractory
	Ulixertinib	NCT03698994	Relapsed/refractory
mTOR	Samotolisib	NCT03213678	Relapsed/refractory
	Sirolimus	NCT02574728	Relapsed/refractory
	Temsirolimus	NCT02389309	Relapsed/refractory
	ABI-009	NCT02975882	Relapsed/refractory
CDK4/6	Palbociclib	NCT03709680	Relapsed/refractory
	Abemaciclib	NCT04238819, NCT02644460	Relapsed/refractory
	Ribociclib	NCT05429502	Relapsed/refractory

Clinicaltrials.gov (accessed on 10 June 2023).

**Table 2:** FDA-approved drugs that are used in the treatment of relapsed high-risk neuroblastoma.

Molecular Target	Drug	Year Approved
ALK	Crizotinib	2011
	Lorlatinib	2018
	Alectinib	2015
	Ceritinib	2019
TRK/ROS1/ALK	Entrectinib	2019
GD2	Naxitimab	2020
	Dinutuximab	2015
	Dinutuximab beta *	2017
RAS-MAPK	Selumetinib	2020
	Binimetinib	2018
mTOR	Sirolimus	1999
	Temsirolimus	2007
CDK4/6	Palbociclib	2015
	Abemaciclib	2017
	Ribociclib	2017

* European Medical Association approval.
